# Phylogenetic diversity of 200+ isolates of the ectomycorrhizal fungus *Cenococcum geophilum* associated with *Populus trichocarpa* soils in the Pacific Northwest, USA and comparison to globally distributed representatives

**DOI:** 10.1371/journal.pone.0231367

**Published:** 2021-01-06

**Authors:** Jessica M. Vélez, Reese M. Morris, Rytas Vilgalys, Jessy Labbé, Christopher W. Schadt

**Affiliations:** 1 Biosciences Division, Oak Ridge National Laboratory, Oak Ridge, TN, United States of America; 2 The Bredesen Center for Interdisciplinary Research and Graduate Education, University of Tennessee, Knoxville, TN, United States of America; 3 Biology Department, Duke University, Raleigh, NC, United States of America; 4 Dept of Microbiology, University of Tennessee, Knoxville, TN, United States of America; Friedrich Schiller University, GERMANY

## Abstract

The ectomycorrhizal fungal symbiont *Cenococcum geophilum* is of high interest as it is globally distributed, associates with many plant species, and has resistance to multiple environmental stressors. *C*. *geophilum* is only known from asexual states but is often considered a cryptic species complex, since extreme phylogenetic divergence is often observed within nearly morphologically identical strains. Alternatively, *C*. *geophilum* may represent a highly diverse single species, which would suggest cryptic but frequent recombination. Here we describe a new isolate collection of 229 *C*. *geophilum* isolates from soils under *Populus trichocarpa* at 123 collection sites spanning a ~283 mile north-south transect in Western Washington and Oregon, USA (PNW). To further understanding of the phylogenetic relationships within *C*. *geophilum*, we performed maximum likelihood and Bayesian phylogenetic analyses to assess divergence within the PNW isolate collection, as well as a global phylogenetic analysis of 789 isolates with publicly available data from the United States, Japan, and European countries. Phylogenetic analyses of the PNW isolates revealed three distinct phylogenetic groups, with 15 clades that strongly resolved at >80% bootstrap support based on a *GAPDH* phylogeny and one clade segregating strongly in two principle component analyses. The abundance and representation of PNW isolate clades varied greatly across the North-South range, including a monophyletic group of isolates that spanned nearly the entire gradient at ~250 miles. A direct comparison between the *GAPDH* and ITS rRNA gene region phylogenies, combined with additional analyses revealed stark incongruence between the ITS and *GAPDH* gene regions, consistent with intra-species recombination between PNW isolates. In the global isolate collection phylogeny, 34 clades were strongly resolved using Maximum Likelihood and Bayesian approaches (at >80% MLBS and >0.90 BPP respectively), with some clades having intra- and intercontinental distributions. Together these data are highly suggestive of divergence within multiple cryptic species, however additional analyses such as higher resolution genotype-by-sequencing approaches are needed to distinguish potential species boundaries and the mode and tempo of recombination patterns.

## Introduction

Plant-fungal relationships are often difficult to disentangle. A single plant species may be associated with hundreds of fungal species and each of these associations can have varying influences on host plant survival and growth that co-vary with the environmental and physical conditions of soil [1–4]. The complexity of these plant-fungal relationships may be at least partially illuminated through targeted understanding of the interactions of exemplar fungal species. Such model species can serve as representatives for exploring plant-fungal interactions and characteristics across various conditions. The genus *Populus* is an excellent plant model for such studies because *Populus trichocarpa* has a fully sequenced genome [5,6] and hosts a diverse community of microbes including bacteria, archaea and fungi [3,7,8] which are capable of accessing, metabolizing, producing, and/or immobilizing compounds which the plant cannot [9]. With over 30 species of deciduous, fast-growing softwoods with distinct sexes and natural hybridization [10], *Populus* also has great importance in agroforestry industries for pulp and paper products, as well as potential as a bioenergy feedstock [11,12].

Similar to *Populus* spp., a correspondingly robust model ectomycorrhizal (ECM) fungal group for paired studies with *Populus* should be widespread, interact with many plant hosts, and be easily culturable in a laboratory setting. The ECM fungal species *Cenococcum geophilum* is a ubiquitously distributed fungus which is positively associated with plant health, growth, and increased soil contaminant resistance [13,14], and as such has the potential to serve well as a model organism for genetic, physiological, and ecological studies. *C*. *geophilum* is known to associate with both angiosperm and gymnosperm species across 40 plant genera, representing over 200 host species [15], and is generally tolerant across wide salinity gradients [16], under water stress conditions [17], and even across extreme soil contamination conditions [16–18]. This wide-ranging resistance to stress conditions in the soil environment is often associated with a high melanin content [16,17,19,20] which may also contribute to hyphal and ECM longevity and resistance to decomposition in soils [21]. *C*. *geophilum* is readily culturable in a laboratory setting and capable of growing on multiple standard solid media, including both defined media such as Modified Melin-Norkrans (MMN) and complex media such as potato dextrose agar (PDA). Additionally, laboratory methods for targeted and relatively rapid isolation of this fungus from soils via its abundant and phenotypically characteristic sclerotia [22,23] allow for efficient isolation of new strains directly from soil samples. All of these characteristics make *C*. *geophilum* an ideal model species and candidate for population-level genomic studies. However most existing regional-level isolate collections have focused primarily on coniferous species [24–26] rather than angiosperms such as *Populus* and many questions about the taxonomic, evolutionary and phylogenetic trajectory of the group of fungi remain uncertain.

The first sequenced genome of *C*. *geophilum* strain 1.58, isolated from Switzerland, was published in four years ago [27]. This genome is among the largest in the fungal kingdom, with a mapped size of 178 Mbp and a total estimated size of up to 203 Mbp [27,28]. *Cenococcum geophilum* has no documented means of sexual spore production [15,29,30], but is considered asexual species despite high levels of genetic and physiological diversity. Due to high levels of diversity that are reported among *C*. *geophilum* isolates, even within isolates from beneath a single tree [31], *C*. *geophilum* has been suggested to represent a complex of indistinguishable cryptic species [15,26,27,32], with many studies finding significant variation in cultured isolate characteristics and physiology [30]. However, patterns of variation consistent with recombination have also been observed by previous studies, suggesting a cryptic sexual state or other mechanisms for intrapopulation recombination [27,29,30,33,34]. Together these studies have led many to suggest that *Cenococcum* may represent an unknown number of cryptic species on a genetic level [15,30,35]. Further supporting this suggestion, a 2016 multigene phylogeny of a previously characterized *C*. *geophilum* isolate collection [23] revealed a divergent clade that was described as a new taxon called *Pseudocenococcum floridanum* [25]. This discovery highlights the need to further explore the phylogenetic diversity among diverse regional isolates of *C*. *geophilum* in order to better characterize this species and determine several factors, including: 1) Is *C*. *geophilum* is a single highly outcrossed species or a heterogenous group of species? 2) If *C*. *geophilum* represents a variety of species, how diverse are the populations are and what are the patterns of speciation? Finally, 3) is there evidence of intra- or interspecies patterns of recombination?

*Cenococcum geophilum* appears to have a myriad of potential benefits as a model ECM and rhizosphere-associated species. However, this fungus has been difficult to study phylogenetically, and further work is needed to delineate its phylogenetic and functional relationships within what may be a highly heterogenous species complex. Our team has set a long-term goal to build a genetically diverse collection of *C*. *geophilum* isolates from across the *Populus trichocarpa* range, mirroring prior host GWAS population studies which have proven valuable for understanding the biology of these host trees [36–43]. Towards this goal we have isolated 229 new *C*. *geophilum* strains from beneath *P*. *trichocarpa* stands over a range of approximately 283 miles of the host range in the United States Pacific Northwest (PNW) states of Oregon and Washington and confirmed their identity using sequencing of the internal transcribed spacer (ITS) region and the glyceraldehyde-3-phosphate dehydrogenase (*GAPDH*) gene. Maximum likelihood phylogenies of the *GAPDH* gene and ITS region of our PNW collection were compared directly in order to identify potential patterns of intra-species recombination. Furthermore, the *GAPDH* genes of our PNW collection were additionally compared to over 500 *C*. *geophilum* isolates with comparable available data from published studies primarily in the United States, European countries, Japan, and other locations where data were publicly available [15,23–26,35], as well as 16 additional European isolates recently sequenced by JGI and provided by Drs. Francis Martin and Martina Peter (Freitas Pereira et al., 2018 [24]).

## Materials and methods

### Site selection and soil sampling

Primary sampling for our study was carried out over a six-day period in late July of 2016. At each site, three, approximately one-gallon soil samples, were collected directly under *P*. *trichocarpa* with at least 50 yards distance in between each tree. Typically, at least three sampling sites were selected along each watershed starting at the Willamette River in Central Oregon and ranging to the Nooksack river near the Canadian border, along a north-south gradient (i.e. the Interstate 5 corridor). Several watersheds and sites corresponded to those previously sampled for the host (Evans et. al. (2014) [43] where possible to allow for testing of associations with *P*. *trichocarpa* GWAS populations in future studies. Soil samples were collected to approximately a 20 cm depth. A trowel was used to fill one-gallon Ziploc freezer bags which were kept on ice and/or refrigerated at 4°C until analysis. Site soil temperatures were recorded using an electronic thermometer (OMEGA model RDXL4SD). Upon return to the lab, two 15 mL tubes of the 105 samples in 2016 were subsampled for soil moisture, carbon (C), nitrogen (N), and soil elemental characterization. Additionally, several isolates derived from smaller exploratory soil samples from within the southern end of this geographic range (sampled by Drs. R. Vilgalys and C. Schadt) for methods development during the prior year were also included in this study, for a total of 123 soil samples used for isolation attempts.

### Sclerotia separation

Soil samples were prepared using a procedure described by Obase et al. (2014) [23] with modifications. Samples were manually sieved and rinsed with distilled water to retain particles between 2mm and 500 μM, and the resulting slurry allowed to soak in distilled water for 10–30 seconds. Floating debris could be decanted off the top. Portions of the slurry were placed into gridded square Petri dishes (VWR 60872–310) which were partially filled with water and a dissection scope was used to remove sclerotia using tweezers. Sclerotia were submerged in undiluted Clorox bleach for 40 minutes using a VWR 40 μm nylon cell strainer (Sigma-Aldrich Z742102), then rinsed 3 times with sterile distilled water. Sclerotia which did not turn white after bleach treatment were plated onto Modified Melin-Norkrans (MMN) media (after [44] composed of 3 g l^-1^ malt extract, 1.25 g l^-1^ glucose, 0.25 g l^-1^ (NH_4_)_2_HPO_4_, 0.5 g l^-1^ KH_2_PO_4_, 0.15 g l^-1^ MgSO_4_-7H_2_O, 0.05 g l^-1^ CaCl_2_, 1 mL^-1^ FeCl_3_ of 1% aqueous solution, and 10 g l^-1^ agar, adjusted to 7.0 pH using 1N NaOH. After autoclaving media were allowed to cool to 60°C, then 1 g l^-1^ thiamine was added along with the antibiotics Ampicillin and Streptomycin at 100 ppm each. Plates with sclerotia were stored in the dark at 20°C.

### DNA extraction

Isolates with dark black growth were considered viable and allowed to grow at 20°C until ~5 mm diameter (approx. 6 weeks to 3 months). Colonies were transferred onto a cellulose grid filter (GN Metricel 28148–813) on MMN plates using the previous protocol except adding 7 g l^-1^ dextrose and omitting antibiotics and allowed to grow for 1–3 months for DNA extraction. The Extract-N-Amp kit (Sigma-Aldrich XNAP2-1KT) was used to extract genomic DNA, following manufacturer instructions except the modification to use only 20 μL of the Extraction and Dilution solutions [45]. DNA samples were stored at -20°C until use in PCR and sequencing efforts below.

### PCR amplification

Ribosomal DNA (rRNA) was amplified using fungal-specific ITS primers ITS1 and ITS4 [46], and the *GAPDH* gene was amplified using the gpd1 and gpd2 primers [47] using the Promega GoTaq © Master Mix kit to amplify DNA. The thermocycling conditions consisted of an initial hold of 94°C for 5 min, followed by 35 cycles of 94°C (30 s), 55°C (30 s), and 72°C (2 min), with a final elongation of 72°C for 10 min. Amplified PCR products were analyzed on a 1% agarose gel using TAE buffer to confirm band size prior to cleanup. PCR products were then cleaned using the Affymetrix USB ExoSAP-IT © kit and sequenced on an ABI3730 Genetic Analyzer at the University of Tennessee at Knoxville (UTK), or at Eurofins Genomics (Louisville, Kentucky, USA). Sequences generated were analyzed against the NCBI database using the BLAST feature in Geneious version 10.2.3 (https://www.geneious.com, Kearse et al., 2012 [48]) to verify fungal identity as *C*. *geophilum*. Confirmed *C*. *geophilum* isolates (marked “CG” with number) were stored on MMN plates at 20°C and re-plated quarterly to ensure continued viability.

### Determination of native soil properties

For the 105 soil samples collected in July of 2016, soil temperature, concentrations of carbon, nitrogen, elemental metals, and soil water content were analyzed. A C/N analysis was conducted on approximately 18g of each soil sample. The samples were oven-dried at 70°C and ground to a fine powder. Approximately 0.2 g of ground sample were analyzed for carbon and nitrogen on a LECO TruSpec elemental analyzer (LECO Corporation, St. Joseph, MI). Duplicate samples and a standard of known carbon and nitrogen concentration (Soil lot 1010, LECO Corporation, carbon = 2.77% ± 0.06% SD, nitrogen = .233% ± 0.013% SD) were used to ensure the accuracy and precision of the data.

Soil elemental metal concentrations were determined using the Bruker Tracer III-SD XRF device. Approximately 1g of dried, homogenized soil was placed into Chemplex 1500 series sample cups with Chemplex 1600 series vented caps and 6 uM Chemplex Mylar® Thin-Film. Cups were placed against the XRF examination window and scanned for 60 seconds at 40 kV with a vacuum and no filter. Elemental spectra were collected using the Bruker S1PXRF S1 MODE v. 3.8.30 software and analyzed using the Bruker Spectra ARTAX v. 7.4.0.0 software. Correlation coefficients relating the total sclerotia isolated, total *Cenococcum* isolates per site, soil temperature, percent moisture content, C and N weight percent, and total counts per minute (cpm) of a range of elements were then calculated using the R v.3.4.1 statistical analysis software (2017) [49] and corrplot package v. 0.84 (https://cran.r-project.org/web/packages/corrplot/index.html) in order to determine whether relationships existed between soil quality and content and *C*. *geophilum* abundance and isolation success.

### Phylogenetic analyses

The nrITS I and II gene region and *GAPDH* gene sequences of 228 PNW *C*. *geophilum* isolates were successfully amplified and sequenced, trimmed and aligned to the published genome of the *C*. *geophilum* strain 1.58 using Geneious. Data sets were deposited into the NCBI GenBank database and made accessible (S1 Table). The ITS and *GAPDH* phylogenies were concatenated for using Geneious, and isolates lacking either ITS or *GAPDH* sequence data were excluded from the multigene concatenated analysis. For comparison with globally distributed isolates, ITS and *GAPDH* sequence data of 543 *C*. *geophilum* strains were obtained from GenBank including strains from Japan, Europe, the United States, and 16 additional European isolates recently sequenced by JGI were provided by Drs. Francis Martin and Martina Peter (personal communication, Freitas Pereira et al., 2018 [24]). These isolates were aligned with the Pacific Northwest (PNW) isolate collection using ITS and *GAPDH* separately, as well as a multigene concatenation of ITS and *GAPDH*. All phylogenies were rooted using the outgroups *Glonium stellatum*, *Hysterium pulicare*, and *Pseudocenococcum floridanum* isolate BA4b018 [25] obtained from GenBank (https://www.ncbi.nlm.nih.gov/genbank/) or MycoCosm [50,51] (https://genome.jgi.doe.gov/programs/fungi/index.jsf).

The best models for maximum likelihood analyses were determined for each individual gene alignment and concatenated gene region alignments using the Find Best DNA/Protein Models feature in MEGA X [52] to determine the Akaike Information Criterion (AICc) value [53] and Bayesian Information Criterion (BIC) [54], with the best model indicated by the lowest AICc and BIC values (Table 1). A maximum likelihood (ML) phylogenetic analysis was produced for the PNW isolate collection ITS, *GAPDH* and concatenated phylogenies using the MEGA X software with the determined best model settings using 1000 bootstrap replications. Bayesian probabilities of the PNW and global single gene alignments were also inferred using the MrBayes v. 3.2.7a software [55] with the determined best model settings. Isolates lacking either ITS or *GAPDH* sequence data were excluded from the concatenated ML analysis of the global isolate collection for a total of 499 isolates, and the concatenated alignment was partitioned by gene region for analyses. The alignments and phylogenetic tree files are deposited in GitHub and may be accessed at https://github.com/velezjm/cenococcum_phylogenetics.

**Table 1:** Model with best fit analysis, Bayesian Information Criterion, and Akaike Information Criterion (AICc) for each alignment per an analysis using MEGA X software. Lower BIC and AICc values indicate the best model fit for use in analyses.

Alignment	Best Model	AICc	BIC
PNW ITS	K2 + G	2873.433	7082.611
PNW *GAPDH*	K2 + G	5468.328	9860.315
PNW ITS + *GAPDH*	K2 + G	8322.607	12970.723
GP ITS	K2 + G	7258.62	21156.093
GP *GAPDH*	K2 + G	12807.63	29779.549
GP ITS + *GAPDH*	K2 + G + I	15623	26550.792

### Comparison of GAPDH, ITS, and GAPDH + ITS phylogenies

The produced ITS and *GAPDH* ML analyses of the PNW isolate collection were directly compared to both the concatenated phylogeny separately, and to each other, using TreeGraph2 [56] and the R v.3.4.1 statistical analysis software. These analyses indicated a large disagreement between the two gene datasets, more missing taxa, as well as a lack of informative phylogenetic signal/sites from the ITS dataset, and thus only *GAPDH* was used for further phylogenetic relationship inferences as this dataset was the most informative and least confounded.

### Phylogeographic variation within PNW isolates

A correlation plot was created using R statistical software packages Hmisc v. 4.2.0 and corrplot v. 0.84 to determine whether resolved *GAPDH* clades correlated with the latitude of the strain isolation site, and a multiple correspondence analysis (MCA) was conducted to determine clade-specific correlations with latitude. Additionally, a principle components analysis (PCoA) was conducted using a distance matrix generated from the PNW *GAPDH* phylogeny, with and without species outgroups included, in order to determine potential speciation patterns within the PNW isolate collection. The PNW ML analysis was also mapped by isolate site latitude and longitude to the PNW region using the GenGIS 2.5.3 software [57] in order to co-visualize the spatial and phylogenetic diversity within the overall PNW isolates and within resolved clades.

### Global GAPDH phylogenetic analyses

In order to directly compare the new PNW isolates with prior global isolate collections, a global *GAPDH* phylogeny was constructed using a maximum likelihood (ML) analysis in the MEGA X software [52] with 1000 bootstrap replications and the previously determined best fit settings (Table 1). Bayesian probabilities of the global *GAPDH* alignment was also inferred using the MrBayes v. 3.2.7a software [55] with the determined best model settings. Phylogenetic trees were visualized indicating either bootstrap support values or branch lengths using FigTree v. 1.4.4 (http://tree.bio.ed.ac.uk/software/figtree/). Clades were designated as strongly grouped isolates with bootstrap support values of >80%. In addition to avoiding conflicts between *GAPDH* and ITS identified in the PNW isolates, usage of the *GAPDH* rather than multigene concatenated phylogeny allowed for a more comprehensive global isolate collection analysis, as the concatenated global isolate phylogeny excluded >300 isolates which lacked both sequences while the *GAPDH* global isolate phylogeny included a total of 789 isolates. Due to this preponderance of missing ITS data, only the *GAPDH* dataset was used for global collection analyses.

### Recombination patterns within GAPDH and ITS data

The ITS and *GAPDH* RAxML phylogenies were visually compared and analyzed using R package phytools v. 0.6.99. A PCoA was completed for the ITS, *GAPDH*, and concatenated phylogenies and alignments using distance matrices generated using the ape v. 5.3 and seqinr v. 3.6–1 packages in the R statistical software in order to compare patterns of recombination within the PNW isolate collection based on either or both gene regions. Additionally, HKY85 distance matrices were generated using Phylogenetic Analysis Using Parsimony (PAUP) v. 4 [58] and graphed against each other in the R statistical software using the ggplot2 package [59]. The inter-partition length difference (ILD) test was performed on the PNW concatenated alignment, partitioned by gene region, to assess phylogenetic congruence between ITS and *GAPDH* data sets using the PTP-ILD option in PAUP* Version 4 [58] with 1000 permutations and default settings. We additionally used the HKY85 distance matrices to generate cluster distance matrices based on either ITS or *GAPDH* only and then plotted against each other to determine any pattern incongruence between the two genes using the software Mathematica v. 12.0.0.0.

## Results

### A diverse culture collection of PNW Cenococcum isolates associated with Populus

A total of 229 PNW isolates were obtained from 56 out of 123 soil samples (105 primary soil samples from 2016 + 18 preliminary samples from 2015 –S1 Table), accounting for a 46% overall *C*. *geophilum* isolation success rate, as some soil samples had few or no sclerotia. The 105 PNW primary soil samples for which associated soils data were also generated, the total *C*. *geophilum* isolation success rate did not positively correlate to any measured soil condition or quality (S1 Fig). Isolation success also did not strongly correlate to the total number of sclerotia recovered (r = 0.38, p >0.05). Between the soil values however there were expected relationships. For example, the strongest correlation was between the soil C and N weight percentages (r = 0.99, p = <0.05). Strong correlations also existed between the percent moisture content and C content (r = 0.88, p = <0.05) or N content (r = 0.86, p = <0.05), C content and zinc counts per minute (cpm) (r = 0.80, p = <0.05), and N content and zinc cpm (r = 0.81, p = <0.05) suggesting that despite the lack of correlation with sclerotia and isolate numbers our measurement approaches were robust (S1 Fig).

### Phylogeographic variation within PNW isolates

**Fig 1:**
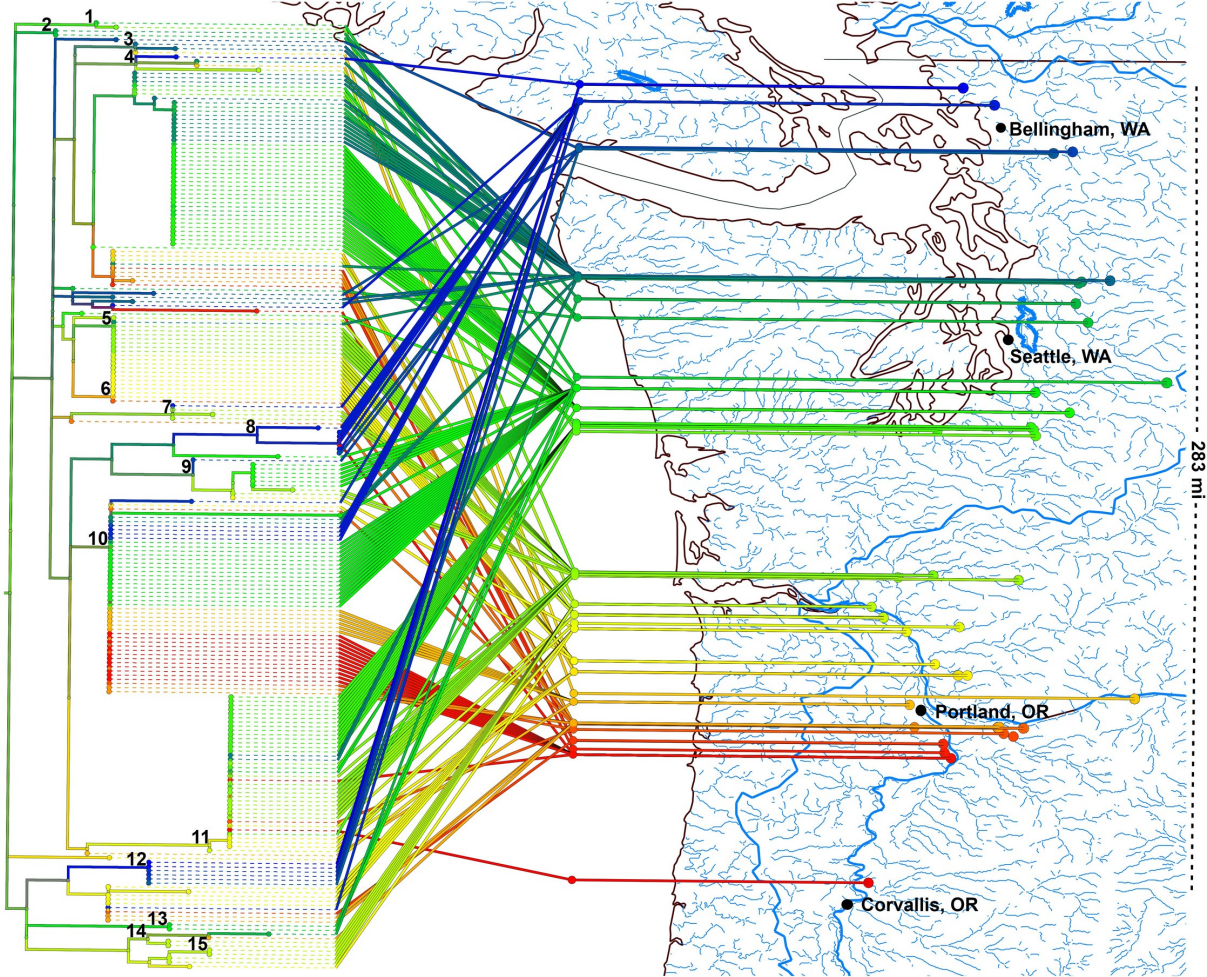
The Pacific Northwest (PNW) isolate *GAPDH* RAxML phylogeny mapped by latitude to the PNW region. Clades with >80% bootstrap support values are indicated above the associated clade. A total of 15 clades encompassing 155 isolates were identified in the PNW isolate collection, with 74 isolates remaining unresolved. Isolates from smaller clades tended to group by region of origin with a few notable exceptions present over large north-south distances.

A total of 438 *GAPDH* positions were represented in the alignment for the *GAPDH* ML analyses of the PNW and global isolate collections. In the PNW isolates, the phylogeny backbone strongly resolved the PNW collection at 97.1%. A total of 155 isolates grouped into 15 clades where two or more strains were resolved at >80% bootstrap values and 1.0 posterior probability apart from clade 10 (0.8 posterior probability) (Fig 1, S2 Table). Of the 15 resolved clades, two contain nested clades of two or more isolates which resolved at >80% bootstrap support and 1.0 posterior probability (S2 Table). Nonetheless, 74 of our 229 PNW *Populus* isolates were not strongly resolved by these analyses. Within these 15 *C*. *geophilum* clades resolved, across the 283 mile transect of our sites selected along rivers in the PNW, smaller clades tended to group latitudinally by site and watershed of origin, although notable exceptions exist, with two groups containing numerous nearly identical isolates despite distances of >100 miles between their sites of origin. In the PNW isolate collection, 74 isolates were outside of any other strongly supported clades (Fig 1, S2 Table), but the majority grouped strongly (1.0 posterior probability and >80% bootstrap).

**Fig 2:**
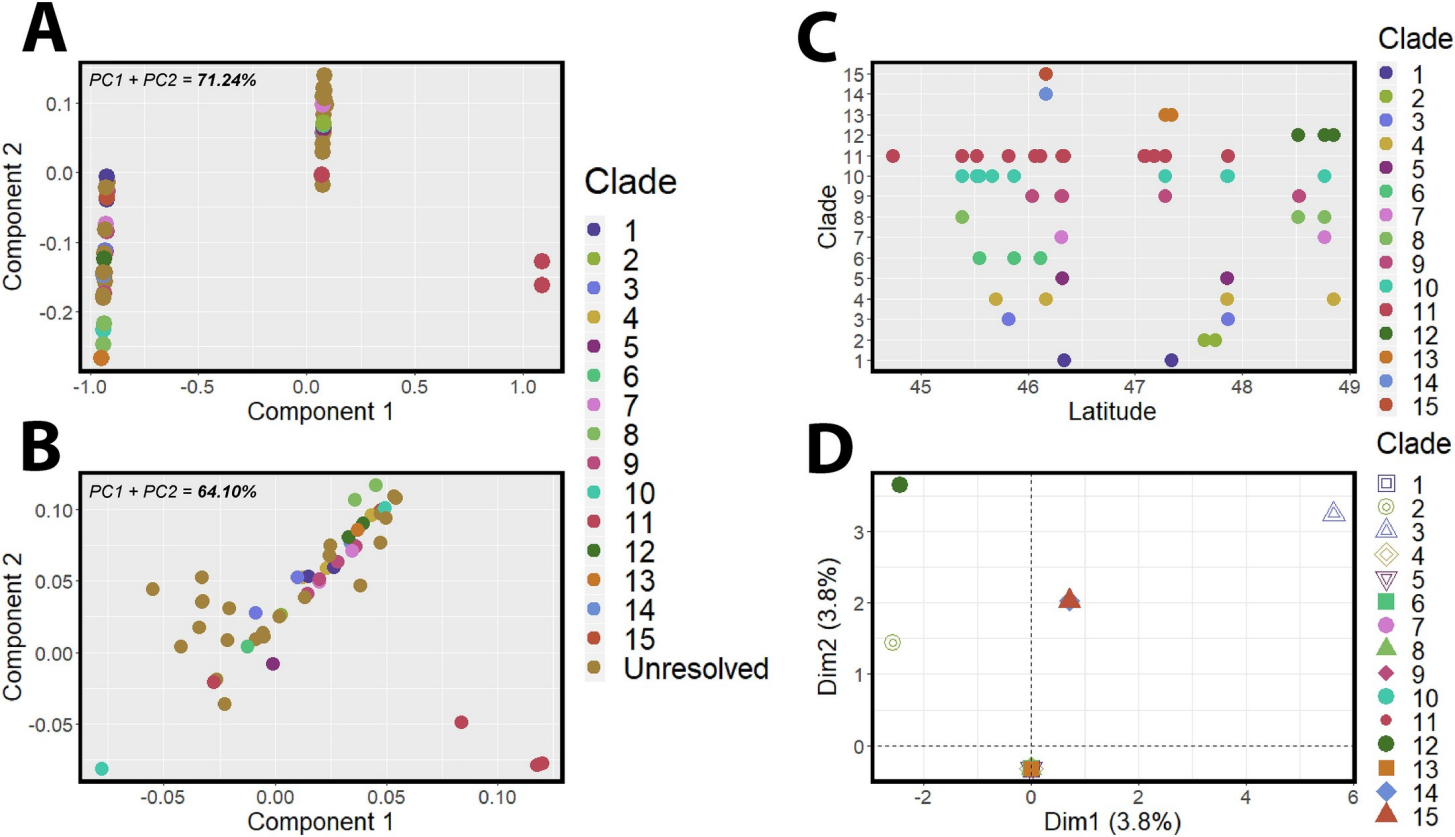
Principle components analysis (PCoA) of the PNW *GAPDH* RAxML phylogeny revealed three distinct isolate clusters, with clade 11 segregating as a separate cluster from the remaining PNW collection (A). A PCoA of the PNW *GAPDH* alignment also revealed three distinct clusters, with clades 10 and 11 segregating from the remaining PNW collection (B). A scatterplot of clade versus latitude did not reveal distinct patterns within the larger groups of the PNW collection (C), and a multiple components analysis showed some differentiation with clades 3, 12, 14, and 15 from the remaining PNW isolate collection, but revealed weak associations overall between latitude, isolate clade, and phylogenetic differentiation (D).

Phylogenetic distance matrices analyzed via PCoA revealed that the PNW isolates group separately as three distinct groups. When using a distance matrix based on the RAxML phylogeny, the PNW 11 clade segregates as a distinct phylogenetic group (Fig 2A), and when using an alignment-generated distance matrix, clades 10 and 11 both segregate as distinct phylogenetic groups (Fig 2B).

The *GAPDH* phylogenetic analysis mapped to the PNW soil sampling sites revealed that isolates did not appear to strongly correlate to original isolation site latitudes (Fig 1, S2 Table). For example, high geographic latitude variation is seen within clade 11, which includes 38 strains isolated >60 miles apart (Fig 1, S2 Table). Clades three, four, five, seven, and nine include strains isolated >100 miles apart (Fig 1, S2 Table). The nested clade 8.1 has the largest spatial range, with a single Willamette river isolate WI7_83.9 grouping with several isolates from sites >200 miles to the north (Fig 1, S2 Table) on the Nooksack River near the Canadian border and the northern end of our transect. Clade ten is the largest clade with a total of 45 isolates (Fig 1, S2 Table), representing the greatest spatial diversity between individual sites within a single clade. The direct mapping to the origin of isolation latitude revealed no clear patterns of segregation, particularly in the largest clades. Further supporting this lack of association with latitude, an MCA found that the combination of clade and latitude were only weakly associated with phylogenetic variation within the PNW isolate collection, with the combination of these two factors accounting for only 7.6% of the phylogenetic variation observed (Fig 2D). Analyses further revealed that clades three, 12, 14, and 15 were not strongly grouped with the majority of the PNW isolate collection (Fig 2D).

### Recombination analyses in the PNW isolate collection

**Fig 3:**
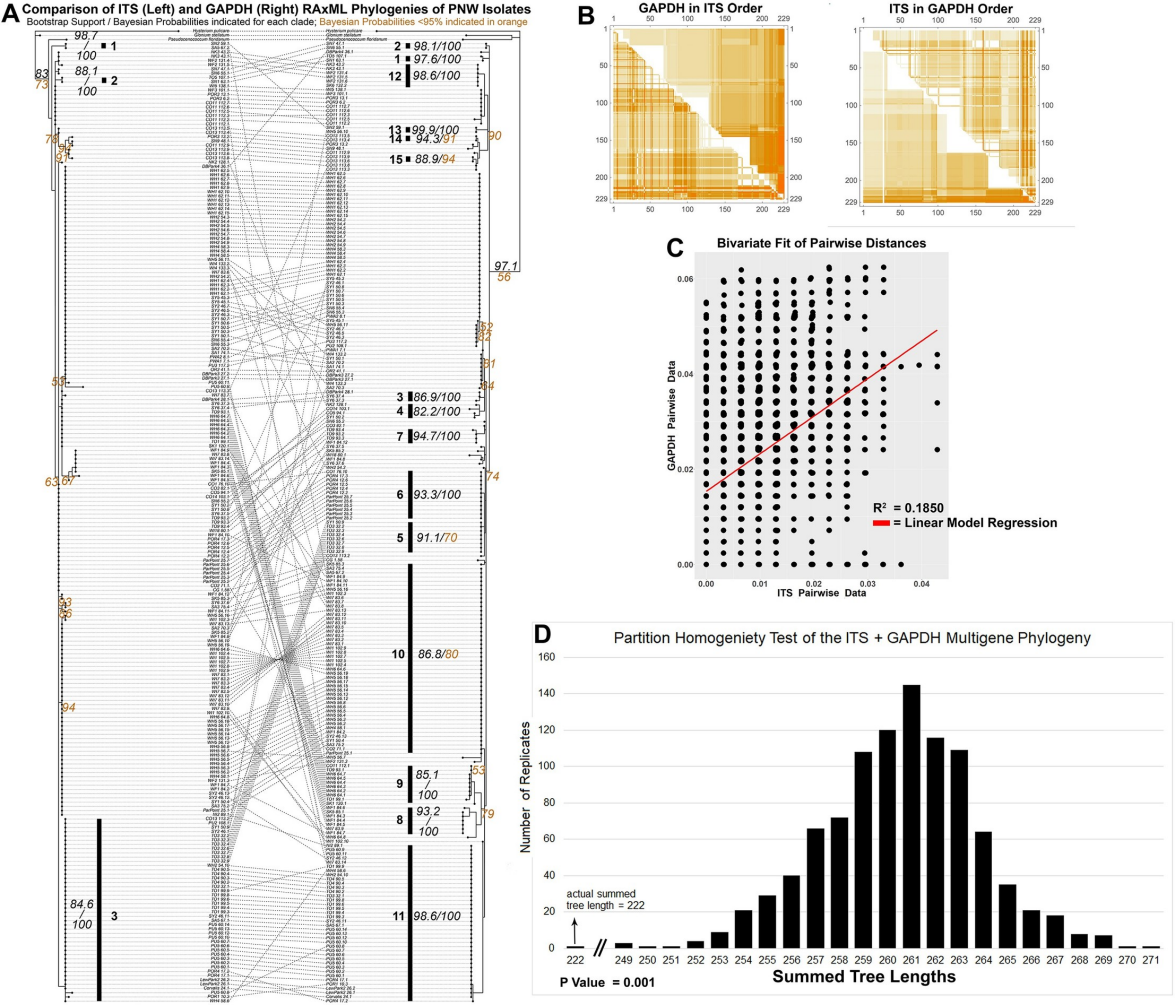
Phylogenetic incongruence between the ITS (left) and *GAPDH* (right) RAxML phylogenies (A). Heatmap showing pairwise distances for ITS and GAPDH genes from *Cenococcum geophilum*. Genetic distances (HKY85) were calculated among all sequence pairs for ITS (above diagonal) and GAPDH (below diagonal). Darker color indicates higher sequence divergence. The first is sequence order based on UPGMA clustering of ITS distances, the second is sequence order based on UPGMA clustering of GAPDH distances (B). A scatterplot of pairwise HKY85 distances for ITS vs *GAPDH* datasets shows low correlation between the ITS and *GAPDH* gene regions (C). The parsimony-based inter-partition length difference (PTP-ILD)test indicated that the ITS and *GAPDH* data sets are incongruent due to no significant difference between the observed data set parsimony trees and the distribution of the randomized tree length calculations (p = 0.001) (D).

While both the ITS and *GAPDH* phylogenies of the PNW isolates had a strongly resolved backbone (>80% bootstrap support), the PNW *GAPDH* phylogeny strongly resolved 15 clades within the PNW isolate collection while the PNW ITS phylogeny only strongly resolved only 3 clades and showed numerous apparent conflicts (Fig 3A). Individually the HKY85 pairwise genetic distances of each gene indicated strong hierarchical clustering, but when plotted against each other that pattern is lost (Fig 3B) and the correlation was observed between the ITS and *GAPDH* gene regions using a linear regression analysis, while significant, was only poorly predictive (R^2^ = 0.185, p < .001, Fig 3C). To further explore this apparent incongruence between the two genes, a total of 102 informative sites of the concatenated PNW alignment were analyzed using the ILD test protocol in PAUP. The ILD test confirmed that the ITS and *GAPDH* data sets are incongruent (Fig 3D), as the summed lengths of the two parsimony trees made from the observed data set were significantly shorter than the distribution of combined tree lengths calculated for 1000 randomizations of the data set (p = 0.001).

### Phylogeographic variation within the global C. geophilum isolate collection

**Fig 4:**
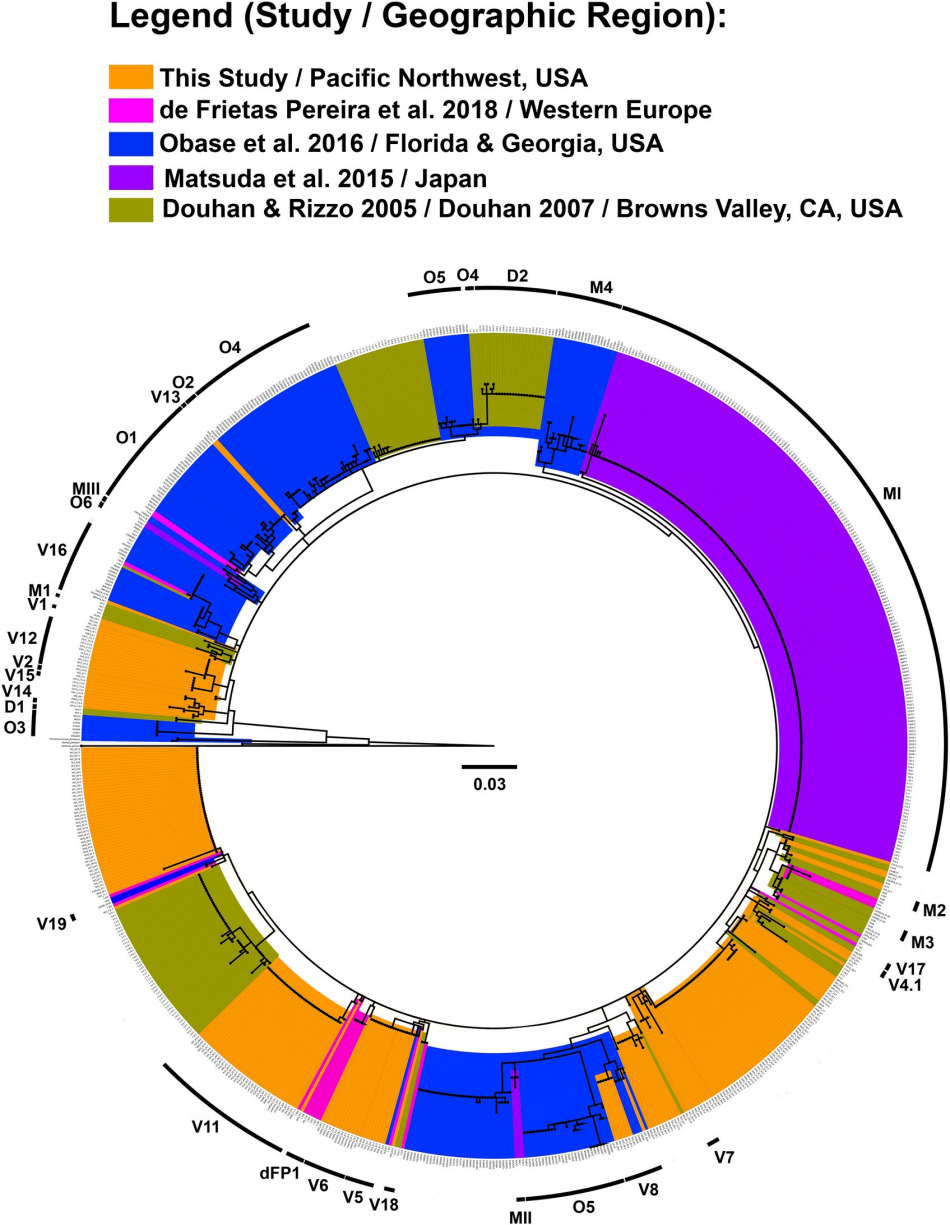
Global isolate collection *GAPDH* RAxML phylogenetic tree. Clades with strong bootstrap support (>80%) are labeled on the outer ring. Strongly supported clades implicated in this study are highlighted in orange and designated with V. Clades V16-V19 represent newly designated clades within the global *C*. *geophilum* isolate collection. The PNW isolates are highlighted in orange. Isolates are highlighted per the most recent published study of origin as follows: D [15,35] highlighted in gold; dFP [24] highlighted in pink; M [26] highlighted in purple; and O [25] highlighted in blue.

Phylogenetic analyses of the PNW isolate collection together with the larger global isolate collection revealed 34 clades of two or more strains resolved at >80% bootstrap support and >0.90 Bayesian posterior probability (Fig 4, S3 Table). The major PNW clades 1, 2, 5, 6, 7, 11, 12, 13, 14, and 15 grouped identically in the global *GAPDH* analysis (clades V1, V2, V5, V6, V7, V11, V12, V13, V14, and V15 respectively (Fig 4, S3 Table). The PNW clade 8 persisted as clade V8 and additionally included isolates CAA022, CAA006, CLW001 and CLW033 from the Florida/Georgia region [25] (Fig 4, S3 Table).

## Discussion

### Cenococcum phylogeographic diversity in the Pacific Northwest

This study is the first comprehensive look at the genetic relationships within a regional population of *Cenococcum geophilum* isolates associated with a single host tree *Populus trichocarpa*. Previous *C*. *geophilum* genetic studies have primarily focused on gymnosperm species such as pines and Douglas fir [25,26], with two studies incorporating isolates collected under an angiosperm (oak) as well as other local gymnosperm host species [15,35] and a second incorporating isolates collected under *Fagus sylvatica* [24] (Table 2). Interestingly, the genetic diversity encountered within this isolate collection exceeds the diversity previously observed in gymnosperm-associated populations by over twofold (Table 2) (Matsuda et al., 2015 [26]; Obase et al., 2016 [25]) implying that *P*. *trichocarpa* may exert fundamentally different selective pressures on the ECM *C*. *geophilum* than gymnosperm hosts.

**Table 2:** Previously resolved clades per study, number of isolates, geographic region, and host plant. The Pacific Northwest (PNW) isolate collection resolved 15 clades which appear to be uniquely associated with *Populus trichocarpa* within its host range in the PNW.

Study	Geographic Region	Total No. Isolates	Host Plant	Gene(s) Used for Phylogenetic Analyses	No. clades resolved at >80% bootstrap support
Douhan & Rizzo 2005 [30]	Browns Valley, California Non-California (location not included)	1037	Oak Not indicated	*GAPDH*	3
Douhan & Huryn 2007 [15]	Browns Valley, California Pacific Northwest, USA Alabama, USA Alaska, USA Europe	74	Mixed host species	*GAPDH*	9^c^
Matsuda et al. 2015 [26]	Japan	225	*Pinus thunbergii*	*GAPDH*	3
Obase et al. 2016 [25]	Florida and Georgia, USA Japan Europe North America	242^a^	*Pinus elliotii* *Pinus taeda* Mixed host species	ITS^b^ *GAPDH*^b^	7^d^
de Freita et al. 2018 [24]	Europe	16	*Picea abies* *Pinus sylvestris* *Fagus sylvatica* Mixed forest	ITS^*b*^ *GAPDH*^*b*^	3^e^
PNW Collection	Pacific Northwest, USA	231	*Populus trichocarpa*	*GAPDH*	15
Global Collection	Pacific Northwest, USA California, USA Florida and Georgia, USA Japan Europe Other North American sites	790	Mixed host species	*GAPDH*	34

a: Representative isolates of 768 total collected from numerous studies.

b: Concatenated.

c: "…a high level of bootstrap support was not found for the majority of the backbone of the phylogeny and thus phylogeographic inference could not be made." (Douhan & Huryn, 2007 [15]).

d: Clade 7 is delimited as the novel species Psuedocenococcum floridanum.

e: Clades previously indicated in Obase et al. 2016 [25].

While geographic patterns are clearly evident within the phylogenetic analysis of our PNW isolates, interestingly the soil variables examined seemed to have no correlation with either the number of *C*. *geophilum* sclerotia recovered, the number of isolates obtained from our samples or their phylogenetic relationships (S1 Fig). Other researchers have previously found aluminum concentrations to be related to the formation and abundance of sclerotia, primarily in pine forests [60–62], however none of the soil chemistry data was relatable to the isolate diversity within or between our sites. We also observed a much lower abundance of *Cenococcum* sclerotia under *Populus trichocarpa* hosts than reported for other systems. Our methods were only semi-quantitative as we were focused on diversity rather than biomass, however we were typically only able to recover less than 20 sclerotia, and in approximately half our samples zero sclerotia, per gallon (~3.5 kg) of soil sampled. While much of the literature uses similar semi-quantitative methods, one previous study of Douglas Fir forests also conducted in western Oregon, averaged 0.91 sclerotia per gram of soil [63] translating to 2785 kg ha^-1^ of *Cenococcum* sclerotial biomass. This level of abundance associated with a different host in the same region, would seem to be approximately two orders of magnitude greater than those recovered in our study under *Populus*.

### Incongruent genes and indications of recombination within the PNW isolate collection

A side by side comparison of the ITS and *GAPDH* phylogenies showed many inconsistencies. For example in some cases up to three ITS types associated with a single *GAPDH* gene group, and numerous groupings in the respective gene phylogenies were inconsistent with each other. While processes such as homoplasy can lead to such inconsistency patterns in ancestral lineages, this pattern was also present in more terminal branches which could be suggestive of ongoing or historic intra- or inter-species recombination events among the PNW isolates (Fig 3A), and prompted further analyses. While the HKY85 pairwise genetic distances of each gene individually reveal clear hierarchical clustering, when plotted against each other that pattern is lost (Fig 3B), further supporting demonstrating a high level of incongruence between the two genes and a linear analysis indicated no relationships between pairwise distances within the *GAPDH* and ITS datasets (Fig 3C). An inter-partition length difference test [58,64] also confirmed that the *GAPDH* and ITS data are incongruent (Fig 3D), indicating that the two genes have conflicting phylogenetic history or else are otherwise drawn from different distributions. These data may reflect previous evidence of cryptic recombination observed in *C*. *geophilum* in some of the first studies of this species that used phylogenetic approaches over 20 years ago [34] and are largely consistent with studies using a variety of methods both in *Cenococcum* [15,30,32,33] and other ascomycetes [65,66]. Similarly, our study suggests that recombination could have been active in the PNW isolate collection and is reflected in the incongruent histories of inheritance between the ITS and *GAPDH* gene regions. However, while the history of these two genes show patterns consistent with recombination, only two genes were investigated which greatly limits the strength of our inferences. Gaining evidence across many more loci within the population will be important to verify these patterns and fully quantify the rate and extent of gene flow.

Incomplete lineage sorting is also an unlikely explanation for the patterns in our analysis within this population, as the comparison of nrITS and *GAPDH* phylogenies suggests many recombination events. Additionally the ILD test, which represents a minimum estimate to rectify the phylogenies, suggests that there were possibly many events, some of which are found deep within the phylogeny (Fig 3A–3D). Of course, a high level of incongruence between these two genes is not completely conclusive. Additional genomic sequencing could further elucidate the likelihood of recombination versus incomplete lineage sorting. Genes such as ITS are known to undergo concerted evolution after hybridization, so we would not expect ITS to accurately reflect the history of any rare recombination events. While the ITS region is used as the universal standard for fungal species identification [67], intragenomic variation also presents serious concerns in some cases, where disparate copies may be found within the genome of individual isolates [68,69]. Previous studies have also observed that both closely related and cryptic species are difficult to delineate based on ITS region phylogenetic analyses [70,71], and the presence of multiple copies of the ITS region within nuclear genomes increases this risk of inaccurate delineation [69,72]. As a result, the GAPDH gene is considered more phylogenetically informative in such analyses [26]. This discrepancy in apparent rates of recombination is consistent with the increased grouping shown by *GAPDH* (Fig 3A), which as a single copy gene is not subject to the same limitations as ITS. Ultimately, regardless of the causal evolutionary mechanism, our comparison of the ITS and *GAPDH* gene phylogenies showed incompatible modes and tempo of evolutionary change between the multi-copy nrITS region and the single-copy protein-coding *GAPDH* gene in these taxa, which at minimum suggest caution in interpreting similar studies where these genes are concatenated for analysis.

### Cenococcum phylogeographic diversity within a global context

Our study further increases the known diversity of *C*. *geophilum* within the global isolate collection, with many of the PNW isolate collection clades appearing to be unique when compared to those previously reported (Fig 4). Distinct groups that were present in previous analyses are indicated using the last initial of the first author of the original study (Fig 4, S3 Table). Four newly designated clades, V16-V19, represent never-before-seen relationships determined through a ML and Bayesian analysis using best fit parameters (Fig 4, S3 Table). One of these clades, V16, includes isolates from both the North American and European continents, and both clade V8 and V17 include isolates from both the west and east coasts of the United States (Fig 4, S3 Table). The genetic diversity of our *C*. *geophilum* isolates from our Pacific Northwest sampling also appears greater in comparison to that found in other regions, with previous studies resolving a maximum of 9 clades within any particular collection [15,24–26,35] (Table 2).

Four of the identified clades in the PNW isolates grouped similarly between our PNW and global phylogenies, but remained unaffiliated with any other isolates in the global study, implying that clades three, four, and nine may be specifically associated with *Populus* in the Pacific Northwest. Alternatively these “core” *C*. *geophilum* isolates may be associated with other hosts that are perhaps not well represented in the rather sparse global samplings to date. Additionally, our analyses suggest PNW clades 10 and particularly 11 may represent particularly divergent clonal groups or incipient species within the PNW isolate collection, as both clades segregated strongly from the rest of the PNW isolate collection and other global representatives.

A 2005 study completed on a collection of *C*. *geophilum* isolated from Browns Valley, CA in the United States based on *GAPDH*, showed a total of three clades identified at greater that 90% bootstrap support [35]. While isolates from this study collection tended to group together in our analyses as well, the associated clades did not persist in the global analysis save for one nested grouping in clade III, designated here as D2 (Fig 4, S3 Table), which includes 23 isolates at a bootstrap support value of 98.3% and posterior probability of 1.0. The identified Obase et al. clade 7 (O7) was described as the new species designated as *Pseudocenococcum floridanum* gen. et sp. nov. We used this taxon as an outgroup for both the PNW and global studies, and none of our isolates appear to be phylogenetically affiliated with *P*. *floridanum* (Fig 4). The isolate sequences provided by Drs. Francis Martin and Martina Peter generally remained unresolved in the global isolate collection analysis with few exceptions. One such exception, clade dFP1, includes four isolates from Switzerland and mirrors the phylogenetic relationship observed in de Freitas Pereira et al. (2018) [24] (Fig 4, S3 Table). However from our analyses, it appears European sites may remain under-sampled overall.

Two of the newly designated clades, V16 and V17, encompass the greatest geographic diversity in the global isolate collection analysis. The clade V16 includes 22 intercontinental total isolates grouped into 4 strongly supported nested clades (>90% bootstrap value, 1.0 posterior probability) (Fig 4, S3 Table), and Clade V17 (>90% bootstrap value, 1.0 posterior probability) includes North American cross-continental isolates from Browns Valley, CA, US, and Florida/Georgia (Fig 4, S3 Table). These large spatial distances across which several well-resolved clades were observed within both the PNW and global isolate analyses, highlight the need for higher resolution genetic studies. There is a possibility that greater genetic resolution will subdivide such groups despite spatially distinct origins to strongly group together based on origin. However, the cryptic nature of *C*. *geophilum* also presents the possibility that these wide distribution patterns between isolates will become more extreme as well. Either case will provide further understanding of the patterns of speciation and context for understanding genetic exchange within the *C*. *geophilum* species complex.

### Conclusions and future directions

The genetic diversity present within both local and global isolate collections of *C*. *geophilum* isolates is striking. Our study reveals the existence of multiple cryptic clades of *C*. *geophilum* as well as distinct phylogenetic groups from the PNW which may be uniquely associated with *P*. *trichocarpa*, and confirms the common view of this species as a hyper-diverse group of ectomycorrhizal fungi on both regional and global scales. Our study additionally indicates incongruent patterns of the *GAPDH* and ITS gene regions that may be suggestive of recombination. However, to provide further evidence as to whether this represents one global, hyper-diverse species, or a myriad of cryptic species, a more robust analysis with greater population-level resolution across many loci to more accurately quantify gene flow will be required. Future research could further elucidate these regional and global relationships through the use of genotype-by-sequencing (GBS) or restriction-associated DNA sequencing (RAD-seq) approaches for more analysis [73]. Such approaches could help shed a light on this ubiquitous fungal taxon which has proven historically difficult to classify both physiologically and phylogenetically, and allow us the opportunity to delineate the individual species which may currently be included in this greater *C*. *geophilum* species complex, or the other mechanisms by which it may maintain such diversity.

## Supporting information

S1 Fig Correlogram of PNW clade, rivershed of origin, site latitude, soil percent moisture content, C/N weight percent, temperature, elemental metal (lead, zinc, copper, cadmium, strontium) counts per minute, total sclerotia isolated, and total *C*. *geophilum* isolation success of 105 PNW soil samples. Positive correlations are highlighted in blue and negative correlations are highlighted in red, with color intensity proportional to the correlation coefficient. Only those correlation coefficients with p<0.05 are shown in color. No measured soil conditions or qualities were determined to correlate with the total sclerotia obtained or *C*. *geophilum* successfully isolated.
(TIF)

S1 Table Pacific Northwest isolate sites and total *Cenococcum geophilum* isolated. The overall isolation success rate of *C*. *geophilum* from 105 soil samples was 46%. Total *C*. *geophilum* isolates >0 are bolded in the Total *Cenococcum* column.
(XLSX)

S2 Table Pacific Northwest *Cenococcum geophilum* isolates, rivershed of origin, associated clade and bootstrap support value. Bootstrap values of >85% are bolded. Unresolved isolates or isolates not grouped into a nested clade are designated with a dash (-). The ITS and GAPDH GenBank accession numbers are included.
(XLSX)

S3 Table Global population GAPDH maximum likelihood analysis of 790 *Cenococcum geophilum* strains. Groupings implicated or identified in previous studies are indicate as follows: D [15,35]; dFP [24]; M [26]; and O [25]. Strongly supported clades designated in this study are designated with V. Clades V16-V19 represent newly designated clades within the global *C*. *geophilum* population. Bootstrap support values of >85% are bolded. Isolates not grouped into a nested clade are designated with a dash (-).
(XLSX)

## References

[1] Grayston SJ, Wang SQ, Campbell CD, Edwards AC. Selective influence of plant species on microbial diversity in the rhizosphere. Soil Biology & Biochemistry. 1998 pp. 369–378. 10.1016/s0038-0717(97)00124-7

[2] Raynaud X, Jaillard B, Leadley PW. Plants May Alter Competition by Modifying Nutrient Bioavailability in Rhizosphere: A Modeling Approach. Am Nat. 2008;171: 44–58. 10.1086/523951 18171150

[3] Bonito G, Reynolds H, Robeson MS, Nelson J, Hodkinson BP, Tuskan G, et al Plant host and soil origin influence fungal and bacterial assemblages in the roots of woody plants. Mol Ecol. 2014;23: 3356–3370. 10.1111/mec.12821 24894495

[4] Kennedy PG, Higgins LM, Rogers RH, Weber MG. Colonization-competition tradeoffs as a mechanism driving successional dynamics in ectomycorrhizal fungal communities. PLoS One. 2011;6: e25126 10.1371/journal.pone.0025126 21949867 PMC3176321

[5] Torr P, Putnam N, Ralph S, Rombauts S, Salamov A, Schein J, et al The Genome of Black Cottonwood Populus trichocarpa (Torr. & Gray). Science (80-). 2006;313: 1596–1604. 10.1126/science.1128691 16973872

[6] Tuskan GA, Difazio SP, Teichmann T. Poplar Genomics is Getting Popular: The Impact of the Poplar Genome Project on Tree Research. Plant Biol. 2004;6: 2–4. 10.1055/s-2003-44715 15095128

[7] Cregger MA, Veach AM, Yang ZK, Crouch MJ, Vilgalys R, Tuskan GA, et al The Populus holobiont: dissecting the effects of plant niches and genotype on the microbiome. Microbiome. 2018;6: 31 10.1186/s40168-018-0413-8 29433554 PMC5810025

[8] Hacquard S, Schadt CW. Towards a holistic understanding of the beneficial interactions across the Populus microbiome. New Phytol. 2015;205: 1424–1430. 10.1111/nph.13133 25422041

[9] Berendsen RL, Pieterse CMJJ, Bakker PAHMHM. The rhizosphere microbiome and plant health. Trends Plant Sci. 2012;17: 478–486. 10.1016/j.tplants.2012.04.001 22564542

[10] Jansson S, Bhalerao RP, Groover AT. Genetics and Genomics of Populus. Springer Science + Business Media; 2010.

[11] Porth I, El-Kassaby YA. Using Populus as a lignocellulosic feedstock for bioethanol. Biotechnol J. 2015;10: 510–524. 10.1002/biot.201400194 25676392

[12] Sannigrahi P, Ragauskas AJ, Tuskan GA. Poplar as a feedstock for biofuels: A review of compositional characteristics. Biofuels, Bioprod Biorefining. 2012;4: 209–226. 10.1002/bbb.206

[13] Meena VS, Mishra PK, Bisht JK, Pattanayak A. Agriculturally Important Microbes for Sustainable Agriculture. Springer Nature Singapore; 2017 10.1007/978-981-10-5343-6

[14] Zong K, Huang J, Nara K, Chen Y, Shen Z, Lian C. Inoculation of ectomycorrhizal fungi contributes to the survival of tree seedlings in a copper mine tailing. J For Res. 2015;20: 493–500. 10.1007/s10310-015-0506-1

[15] Douhan GW, Huryn KL, Douhan LI, Mycologia S, Dec NN, Douhan GW, et al Significant diversity and potential problems associated with inferring population structure within the Cenococcum geophilum species complex. Mycologia. 2007;99: 812–819. 10.3852/mycologia.99.6.812 18333505

[16] Matsuda Y, Yamakawa M, Inaba T, Obase K, Ito S ichiro. Intraspecific variation in mycelial growth of Cenococcum geophilum isolates in response to salinity gradients. Mycoscience. 2017;58: 369–377. 10.1016/j.myc.2017.04.009

[17] Fernandez CW, Koide RT. The function of melanin in the ectomycorrhizal fungus Cenococcum geophilum under water stress. Fungal Ecol. 2013;6: 479–486. 10.1016/j.funeco.2013.08.004

[18] Krpata D, Peintner U, Langer I, Fitz WJ, Schweiger P. Ectomycorrhizal communities associated with Populus tremula growing on a heavy metal contaminated site. Mycol Res. 2008;112: 1069–1079. 10.1016/j.mycres.2008.02.004 18692376

[19] Cordero RJB, Casadevall A. Functions of fungal melanin beyond virulence. Fungal Biol Rev. 2017; 1–14. 10.1016/j.fbr.2016.12.003 31649746 PMC6812541

[20] Toledo AV, Franco MEE, Yanil Lopez SM, Troncozo MI, Saparrat MCN, Balatti PA. Melanins in fungi: Types, localization and putative biological roles. Physiol Mol Plant Pathol. 2017 10.1016/j.pmpp.2017.04.004

[21] Fernandez CW, McCormack ML, Hill JM, Pritchard SG, Koide RT. On the persistence of Cenococcum geophilum ectomycorrhizas and its implications for forest carbon and nutrient cycles. Soil Biol Biochem. 2013;65: 141–143. 10.1016/j.soilbio.2013.05.022

[22] Trappe JM. Studies on Cenococcum graniforme. I. an efficient method for isolation from sclerotia. Can J Bot. 1969;47: 1389–1390. 10.1139/b69-198

[23] Obase K, Douhan GW, Matsuda Y, Smith ME. Culturable fungal assemblages growing within Cenococcum sclerotia in forest soils. FEMS Microbiol Ecol. 2014;90: 708–717. 10.1111/1574-6941.12428 25229424

[24] de Freitas Pereira M, Veneault-Fourrey C, Vion P, Guinet F, Morin E, Barry KW, et al Secretome Analysis from the Ectomycorrhizal Ascomycete Cenococcum geophilum. Front Microbiol. 2018;9: 1–17. 10.3389/fmicb.2018.00001 29487573 PMC5816826

[25] Obase K, Douhan GW, Matsuda Y, Smith ME. Revisiting phylogenetic diversity and cryptic species of Cenococcum geophilum sensu lato. Mycorrhiza. 2016;26: 529–540. 10.1007/s00572-016-0690-7 26968743

[26] Matsuda Y, Takeuchi K, Obase K, Ito S. Spatial distribution and genetic structure of Cenococcum geophilum in coastal pine forests in Japan. FEMS Microbiol Ecol. 2015;91: fiv108 10.1093/femsec/fiv108 26347080

[27] Peter M, Kohler A, Ohm RA, Kuo A, Krützmann J, Morin E, et al Ectomycorrhizal ecology is imprinted in the genome of the dominant symbiotic fungus Cenococcum geophilum. Nat Commun. 2016;7: 12662 10.1038/ncomms12662 27601008 PMC5023957

[28] Talhinhas P, Tavares D, Ramos AP, Gonçalves S, Loureiro J. Validation of standards suitable for genome size estimation of fungi. J Microbiol Methods. 2017;142: 76–78. 10.1016/j.mimet.2017.09.012 28923689

[29] Jany J, Garbaye J, Martin F. Cenococcum geophilum populations show a high degree of genetic diversity in beech forests. New Phytol. 2002;154: 651–659. 10.1046/j.1469-8137.2002.00408.x 33873469

[30] Douhan GW, Martin DP, Rizzo DM. Using the putative asexual fungus Cenococcum geophilum as a model to test how species concepts influence recombination analyses using sequence data from multiple loci. Curr Genet. 2007;52: 191–201. 10.1007/s00294-007-0150-1 17768627

[31] Bahram M, Põlme S, Kõljalg U, Tedersoo L. A single European aspen (Populus tremula) tree individual may potentially harbour dozens of Cenococcum geophilum ITS genotypes and hundreds of species of ectomycorrhizal fungi. FEMS Microbiol Ecol. 2011;75: 313–320. 10.1111/j.1574-6941.2010.01000.x 21114502

[32] Obase K, Douhan GW, Matsuda Y, Smith ME. Progress and Challenges in Understanding the Biology, Diversity, and Biogeography of Cenococcum geophilum Biogeography of Mycorrhizal Symbiosis. Springer International Publishing; 2017 pp. 299–317. 10.1007/978-3-319-56363-3

[33] Bourne EC, Mina D, Gonçalves SC, Loureiro J, Freitas H, Muller LAH. Large and variable genome size unrelated to serpentine adaptation but supportive of cryptic sexuality in Cenococcum geophilum. Mycorrhiza. 2014;24: 13–20. 10.1007/s00572-013-0501-3 23754539

[34] LoBuglio KF, Taylor JW. Recombination and genetic differentiation in the mycorrhizal fungus Cenococcum geophilum Fr. Mycologia. 2002;94: 772–780. 10.1080/15572536.2003.11833171 21156551

[35] Douhan GW, Rizzo DM. Phylogenetic divergence in a local population of the ectomycorrhizal fungus Cenococcum geophilum. New Phytol. 2005;166: 263–271. 10.1111/j.1469-8137.2004.01305.x 15760369

[36] Bdeir R, Muchero W, Yordanov Y, Tuskan GA, Busov V, Gailing O. Genome-wide association studies of bark texture in Populus trichocarpa. Tree Genet Genomes. 2019;15 10.1007/s11295-018-1309-2 30546292 PMC6287713

[37] Zhang J, Yang Y, Zheng K, Xie M, Feng K, Jawdy SS, et al Genome-wide association studies and expression-based quantitative trait loci analyses reveal roles of HCT2 in caffeoylquinic acid biosynthesis and its regulation by defense-responsive transcription factors in Populus. New Phytol. 2018;220: 502–516. 10.1111/nph.15297 29992670

[38] Tuskan GA, Mewalal R, Gunter LE, Palla KJ, Carter K, Jacobson DA, et al Defining the genetic components of callus formation: A GWAS approach. PLoS One. 2018;13: 1–18. 10.1371/journal.pone.0202519 30118526 PMC6097687

[39] Franco-Coronado J, Barry K, Ranjan P, Tuskan GA, Yang Y, Chen J-G, et al Association mapping, transcriptomics, and transient expression identify candidate genes mediating plant–pathogen interactions in a tree. Proc Natl Acad Sci. 2018;115: 11573–11578. 10.1073/pnas.1804428115 30337484 PMC6233113

[40] McKown AD, Klápště J, Guy RD, Soolanayakanahally RY, La Mantia J, Porth I, et al Sexual homomorphism in dioecious trees: Extensive tests fail to detect sexual dimorphism in Populus. Sci Rep. 2017;7: 1–14. 10.1038/s41598-017-01893-z 28500332 PMC5431824

[41] Mckown AD, Klápště J, Guy RD, Geraldes A, Porth I, Hannemann J, et al Genome-wide association implicates numerous genes underlying ecological trait variation in natural populations of Populus trichocarpa. New Phytol. 2014;203: 535–553. 10.1111/nph.12815 24750093

[42] Muchero W, Pryia R, Slavov G, DiFazio S, Schackwitz W, Martin J, et al Populus resequencing: towards genome-wide association studies. BMC Proc. 2011;5: I21 10.1186/1753-6561-5-s7-i21

[43] Evans LM, Slavov GT, Rodgers-Melnick E, Martin J, Ranjan P, Muchero W, et al Population genomics of Populus trichocarpa identifies signatures of selection and adaptive trait associations. Nat Genet. 2014;advance on: 1089–1096. 10.1038/ng.3075 25151358

[44] Marx DH. The influence of ectotrophic mycorrhizal fungi on the resistance of pine roots to pathogenic infections. II. Production, identification, and biological activity of antibiotics produced by Leucopaxillus cerealis var. piceina. Phytopathology. 1969;59: 411–417. 5811914

[45] Kluber LA, Carrino-Kyker SR, Coyle KP, DeForest JL, Hewins CR, Shaw AN, et al Mycorrhizal Response to Experimental pH and P Manipulation in Acidic Hardwood Forests. PLoS One. 2012;7: 1–10. 10.1371/journal.pone.0048946 23145035 PMC3493595

[46] Bokulich NA, Mills DA. Improved selection of internal transcribed spacer-specific primers enables quantitative, ultra-high-throughput profiling of fungal communities. Appl Environ Microbiol. 2013;79: 2519–26. 10.1128/AEM.03870-12 23377949 PMC3623200

[47] Berbee ML, Pirseyedi M, Hubbard S, Pirseyedi M. Cochliobolus Phylogenetics and the Origin of Known, Highly Virulent Pathogens, Inferred from ITS and Glyceraldehyde-3-Phosphate Dehydrogenase Gene Sequences. Mycologia. 1999;91: 964–977.

[48] Kearse M, Moir R, Wilson A, Stones-Havas S, Cheung M, Sturrock S, et al Geneious Basic: An integrated and extendable desktop software platform for the organization and analysis of sequence data. Bioinformatics. 2012;28: 1647–1649. 10.1093/bioinformatics/bts199 22543367 PMC3371832

[49] R Core Team. R: A language and environment for statistical computing R Foundation for Statistical Computing Vienna, Austria: Citeseer; 2017 Available: https://www.r-project.org/.

[50] Grigoriev I V., Nikitin R, Haridas S, Kuo A, Ohm R, Otillar R, et al MycoCosm portal: Gearing up for 1000 fungal genomes. Nucleic Acids Res. 2014;42: 699–704. 10.1093/nar/gkt1183 24297253 PMC3965089

[51] Grigoriev I V., Nordberg H, Shabalov I, Aerts A, Cantor MM, Goodstein D, et al The genome portal of the Department of Energy Joint Genome Institute: 2014 updates. Nucleic Acids Res. 2014;42: 26–32. 10.1093/nar/gkt1069 24225321 PMC3965075

[52] Kumar S, Stecher G, Li M, Knyaz C, Tamura K. MEGA X: Molecular Evolutionary Genetics Analysis across Computing Platforms. Mol Biol Evol. 2018;35: 1547–1549. 10.1093/molbev/msy096 29722887 PMC5967553

[53] Akaike H. Information theory and an extension of the maximum likelihood principle In: Petrov BN, Csaki F, editors. Second International Symposium on Information Theory. Budapest: Akadémiai Kiado; 1973 pp. 267–281. Available: citeulike-article-id:4571969.

[54] Schwarz G. Estimating the Dimension of a Model. Ann Stat. 1978;6: 461–464. 10.1214/aos/1176348654

[55] Huelsenbeck JP, Ronquist F. MRBAYES: Bayesian inference of phylogenetic trees. Bioinformatics. 2001;17: 754–755. 10.1093/bioinformatics/17.8.754 11524383

[56] Stöver BC, Müller KF. TreeGraph 2: combining and visualizing evidence from different phylogenetic analyses. BMC Bioinformatics. 2010;11: 7 10.1186/1471-2105-11-7 20051126 PMC2806359

[57] Parks DH, Mankowski T, Zangooei S, Porter MS, Armanini DG, Baird DJ, et al GenGIS 2: Geospatial Analysis of Traditional and Genetic Biodiversity, with New Gradient Algorithms and an Extensible Plugin Framework. PLoS One. 2013;8 10.1371/journal.pone.0069885 23922841 PMC3726740

[58] Swofford DL. Phylogenetic Analysis Using Parsimony (*and Other Methods). Sunderland, Massachusetts: Sinauer Associates; 2003 Available: https://paup.phylosolutions.com/.

[59] Wickam H. ggplot2: Elegant Graphics for Data Analysis. Verlage, New York: Springer; 2016 Available: https://ggplot2.tidyverse.org.

[60] Watanabe M, Sakagami N, Inoue Y, Genseki A, Ohta H, Fujitake N. The relation between distribution of sclerotium grain and chemical properties in nonallophanic Andosols. Pedologist. 2004;48: 24–32. Available: https://eurekamag.com/research/004/361/004361254.php.

[61] Inoue Y, Kawasaki K, Watanabe M, Ohta H, Bolormaa O, Sakagami N, et al Characterization of major and trace elements in sclerotium grains. Eur J Soil Sci. 2006;58: 786–793. 10.1111/j.1365-2389.2006.00868.x

[62] Watanabe M, Genseki A, Sakagami N, Inoue Y, H. O, Fujitake N. Aluminum oxyhydroxide polymorphs and some micromorphological characteristics in sclerotium grains. Soil Sci Plant Nutr. 2004;50: 1205–1210. 10.1080/00380768.2004.10408595

[63] Fogel R, Hunt G. Fungal and arboreal biomass in a western Oregon Douglas-fir ecosystem: distribution patterns and turnover. Can J For Res. 1979;9: 245–256. 10.1139/x79-041

[64] Burt A, Cartert D a, Koenigt GL, Whites TJ, Taylor JW. Molecular markers reveal cryptic Coccidioides immitis in the human pathogen. Mycotaxon. 1996;93: 770–773. 10.1073/pnas.93.2.770 8570632 PMC40130

[65] Jurado M, Marín P, Vázquez C, González-Jaén MT. Divergence of the IGS rDNA in Fusarium proliferatum and Fusarium globosum reveals two strain specific non-orthologous types. Mycol Prog. 2012;11: 101–107. 10.1007/s11557-010-0733-y

[66] Mirete S, Patiño B, Jurado M, Vázquez C, González-Jaén MT. Structural variation and dynamics of the nuclear ribosomal intergenic spacer region in key members of the Gibberella fujikuroi species complex. Genome. 2013;56: 205–213. 10.1139/gen-2013-0008 23706073

[67] Schoch CL, Seifert K a., Huhndorf S, Robert V, Spouge JL, Levesque C a., et al Nuclear ribosomal internal transcribed spacer (ITS) region as a universal DNA barcode marker for Fungi. Proc Natl Acad Sci U S A. 2012;109: 1–6. 10.1073/pnas.1117018109 22454494 PMC3341068

[68] Simon UK, Weiß M. Intragenomic variation of fungal ribosomal genes is higher than previously thought. Mol Biol Evol. 2008;25: 2251–2254. 10.1093/molbev/msn188 18728073

[69] Kovács GM, Jankovics T, Kiss L. Variation in the nrDNA ITS sequences of some powdery mildew species: Do routine molecular identification procedures hide valuable information? Eur J Plant Pathol. 2011;131: 135–141. 10.1007/s10658-011-9793-3

[70] Stevenson LA, Gasser RB, Chilton NB. The ITS-2 rDNA of Teladorsagia circumcincta, T. trifurcata and T. davtiani (Nematoda: Trichostrongylidae) indicates that these taxa are one species. Int J Parasitol. 1996;26: 1123–1126. 10.1016/S0020-7519(96)80013-0. 8982795

[71] Blouin MS. Molecular prospecting for cryptic species of nematodes: Mitochondrial DNA versus internal transcribed spacer. Int J Parasitol. 2002;32: 527–531. 10.1016/s0020-7519(01)00357-5 11943225

[72] Seifert KA, Wingfield BD, Wingfield MJ. A critique of DNA sequence analysis in the taxonomy of filamentous Ascomycetes and ascomycetous anamorphs. Can J Bot. 1995;73: 760–767. 10.1139/b95-320

[73] Glenn TC, Bayona-Vasquez NJ, Kieran TJ, Pierson TW, Hoffberg SL, Scott PA, et al Adapterama III: Quadruple-indexed, triple-enzyme RADseq libraries for about $1USD per Sample (3RAD). bioRxiv. 2017; 1–34. 10.1101/205799

