# Supplementary figures and images for: Phylogenetic diversity of 200+ isolates of the ectomycorrhizal fungus Cenococcum geophilum associated with Populus trichocarpa soils in the Pacific Northwest, USA and comparison to globally distributed representatives
Source: PLoS One. 2021 Jan 6;16(1):e0231367. doi: 10.1371/journal.pone.0231367 (PMC7787446; doi:10.1371/journal.pone.0231367)

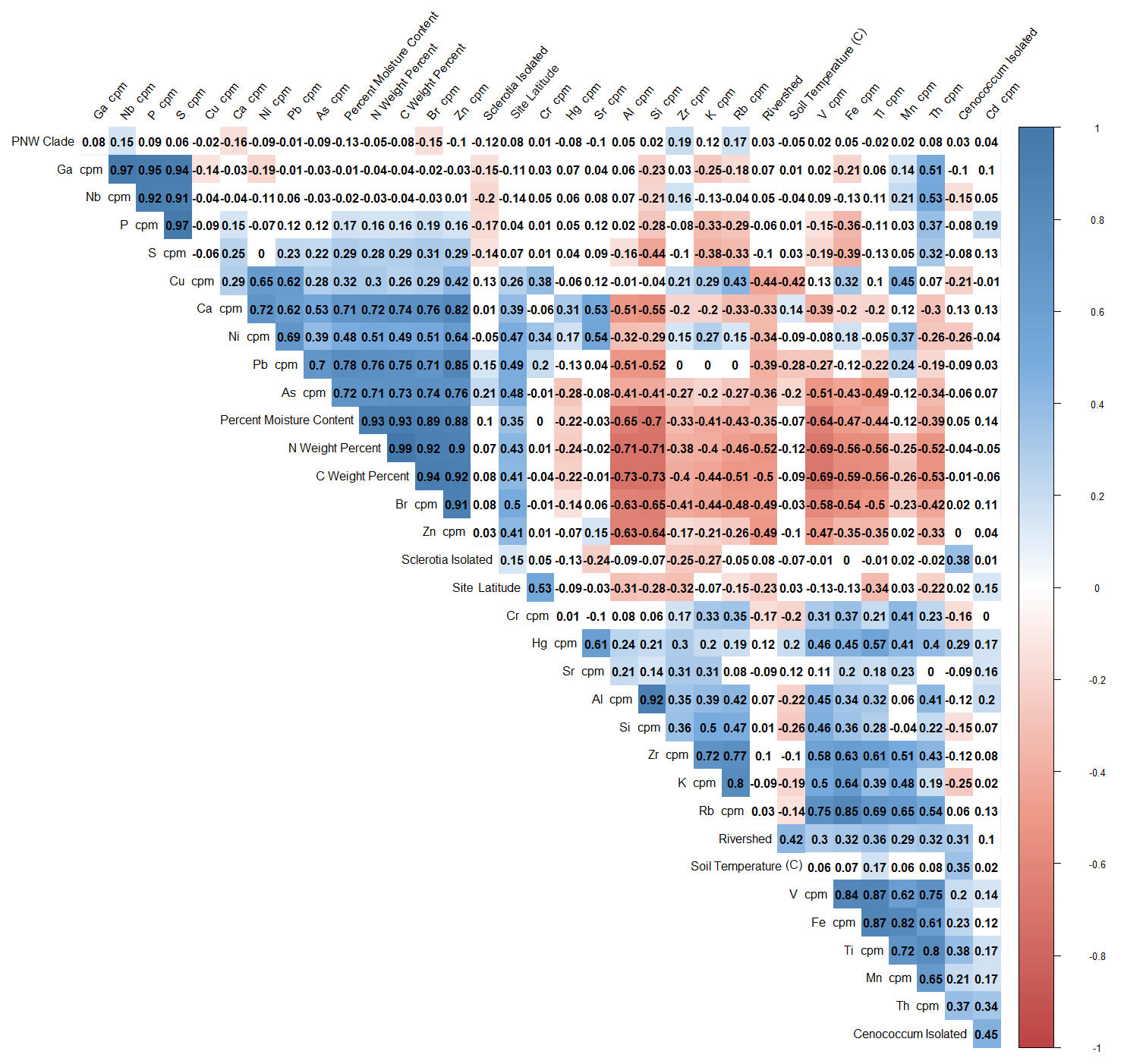

Supplement: S1 Fig — Positive correlations are highlighted in blue and negative correlations are highlighted in red, with color intensity proportional to the correlation coefficient. Only those correlation coefficients with p<0.05 are shown in color. No measured soil conditions or qualities were determined to correlate with the total sclerotia obtained or C. geophilum successfully isolated. (TIF) [file pone.0231367.s001.tif]
